# Leishman’s Midwifery

**Published:** 1874-03

**Authors:** 


### Bibliographical.

---

A SYSTEM OF MIDWIFERY, INCLUDING THE DISEASES OF PREGNANCY AND THE PUERPERAL STATE. *By William Leishman, M.D., Regius Prof. of Midwifery in the University of Glasgow, etc., etc., with* 182 *Illustrations. Philadelphia: Henry C. Lea,* 1873. *Octavo, pp.* 715. *Cloth,* $5.00; *Leather,* $6.00.

This work, a bran new one, fresh from the hand of its author in July last, we take up with mind and heart fully and freely open to the conviction of its merits. In the preface the author boldly announces his purpose to "furnish to students and practitioners a complete system of the midwifery of the present day." How far he has redeemed his pledge, we will endeavor to enable our readers to judge for themselves. Our author in discussing the subject of mestruation remarks, "We are, in the present state of our knowledge, entitled to assume that the periodical discharge depends upon corresponding changes in the ovary, associated with the maturation of a Graafian vesicle." He seems to be ignorant, however, of the results of double ovariotomy as observed by Atlee, Storer, A. Reeves Jackson, and by the writer; not to mention the observation of his own countryman, Dr. Meadows, of London.

The author "disallows" the views and observations of Meigs, Simpson, Murphy and others as to the probability of gestation being, in any case, protracted beyond three hundred days. He informs us that quickening occurs "about the end of the fourth calendar month," and recommends that this observation be compared with the reckoning of 280 days from the last appearance of the menses, to ensure greater accuracy in the prediction of labor. For the vomiting of pregnancy he esteems the effervescing draught of citric acid and bicarbonate potass *combined in the stomach;* he does *not* value the oxalate of cerium of Simpson, but highly, and justly, esteems iced champagne, and thinks pepsin serviceable. Upon the subject of induced abortion he remarks: "We apprehend that when it is a mere question of freeing the woman from the risk of a contingent though not immediate danger, we are in no case warranted in sacrificing the life of the child, and we must, therefore, dissent from the conclusions of those who would sanction such a proceeding in any condition of the mother short of extreme peril.

In plethora threatening convulsions, fearless recourse to blood-letting is enjoined. In albuminuria "antiphlogistic treatment of any kind must be resorted to with the greatest caution." Caution is also given in the use of diuretics. No mention is made of digitalis, which we have found often of great service. The description of the preliminaries to labor is excellent. The instructions given for the management of the third stage of labor are, to our mind, faulty in relying too implicitly upon the efforts of nature, and much unnecessary delay thereby occasioned, to the danger of the parturient patient. The use of the obstetrical bandage is very properly enjoined. A "bolster cover" is recommended; sixteen inches of wide brown cotton-cloth we think decidedly better. The cuts illustrating the mechanism of labor are numerous and very fine; we cannot too warmly express our satisfaction with the illustrations generally throughout the work.

The instructions given for the management of breach labors are, for the most part, both judicious and excellent. There is, however, too much reliance placed upon natural expulsive power for the escape of the head in due season, and the prompt turning out of the head before asphyxia can occur, is not inculcated as forcibly as we think it should be. An illustrative cut exhibits a method of delivery, to which much might be objected.

The author recognizes fully the value of external manipulation upon the abdomen, while the presenting part is elevated by the finger, or fingers, per vaginam, in turning certain cases of preternatural presentations before the waters are drained off. In the treatment of abortion we find no mention made of habitual abortion dependent upon cervical endo-metritis, nor of its prevention by removal of the cause, *i.e.*, by local treatment of the endo-metritis. We have repeatedly carried cases successfully to term in this way who had aborted time after time. The directions for tamponing the vagina are very faulty; surely there is no material at all comparable to raw cotton lint for this use, and Dr. Leishman's recommendation of the obsolete sponge wrung out of vinegar, as used by Dewees, is out of place in a progressive work like this. The placental forceps should be curved to correspond with the arc of Carus, not straight, as represented in the cut.

In placenta prævia the author relies chifly upon turning, and remarks of the tampon, "it is a mere temporary expedient, and, in the case of turning is an almost essential mode of preliminary treatment." He does not approve of extraction of the placenta, excepting under very exceptional conditions, and expresses a very guarded approval of the expedient of separation of the placenta. In post-partum hemorrhage the tampon is condemned and the removal of the clots recommended, but neither in so positive and emphatic terms as they should be; otherwise the injunctions laid down are excellent. The subject of transfusion receives due attention.

While recognizing the differing opinions of others, our author expresses a decided and unqualified preference for the English straight forceps, *i.e.*, those having but one, the cephalic, curve. The reasons given for this preference are, in our opinion, as lame and faulty as is the preference itself. We think no accoucheur who relies exclusively upon the straight forceps can develop in his teachings or practice the full value or capacities of the instrument as an obstetrical aid. His explanation of the mechanical action of the forceps and directions for bringing this mechanical force to bear in the delivery are, to our mind, greatly inferior to those given by Dr. Meigs. An instrument is described and directions given for its use, in *decapitation in utero!*

In executing the operation of turning, "the operator should take off his coat," etc. In company with a distinguished professor of surgery in one of the Scottish Universities, calling at the office of the professor of surgery of an Irish University, we were received by the latter *in his shirt-sleeves*, but with an ample apology. We observed the countenance of our friend to drop at once, and passing out at the door he remarked "I have always taken Prof. —— to be a *nice person;* you perceive how sadly I was mistaken!" Our author esteems it matter of indifference which hand is used in turning. The directions and illustrations for bimanual version are most excellent.

Under the heading of embryotomy, we are counseled: "The conditions which may be held as warranting the operation of embryotomy are those in which the forceps and turning, are of no avail, and which, at the same time, preclude the passage of a living child." If we are to be allowed the *pelvic curve* in our forceps we heartily assent to the dictum, but cannot admit the failure of the *straight* forceps as a ground for embryotomy. The directions given for executing that melancholly, and fortunately, most infrequent, duty of the accoucheur are sufficiently full and complete.

The treatment of the neonatus is well considered, as is also puerperal insanity. In puerperal eclampsia we extract the following opinions and recommendations, viz: "Perhaps, in some quarters, the rejection of the lancet has been too absolute. Indeed, we rather incline to this belief; for there are cases in which the constitution and temperament of the woman, along with the violence of the attack, might lead us, not unnaturally, to suppose that venesection would afford the best chance of recovery." In this sentence we imagine we see evidences of experience and sound judgment struggling to stay, if possible, the flood-tide of popular, professional prejudice. "A remarkable effect is produced, in many cases of puerperal eclampsia, by the administration of chloroform, ether and other anæsthetic agents—an effect which, in some instances, quite surpasses our expectations." "The sedative and narcotic effects of this drug (hydrate chloral) are well known, but it is not so generally understood that when it is pushed further, it produces an anæsthetic effect, under the influence of which a woman may be delivered without experiencing the slightest suffering. We can, without hesitation, corroborate much of what has been advanced of late, in regard to the marvelous effects of this drug in the treatment of convulsive diseases. When given in what we may call ordinary sedative doses, not more than thirty grains—its effect is safe, and in most cases efficacious; but, should we think of giving larger and repeated doses, we should bear in mind that very alarming symptoms are occasionally produced, and that death has even been the result of what we might consider quite an ordinary dose." The propriety of prompt delivery, by the forceps if need be, is enjoined. No allusion is made to the use of veratrum or the bromide potassium. "Should the convulsions persist after delivery, or should they then come on for the first time, full doses of opium or chloral, with cold to the head, perfect rest and quiet, and the emptying of the bowels, if necessary, by a simple enema, are the main points to be attended to. It is very unlikely that at this stage bleeding would be held to be advisable, but it is possible that some benefit might be derived from ligature of the limbs," etc.

In a case where delivery suspended convulsions, which were with difficulty kept under control afterwards by chloroform and bromide, a liberal bleeding from the arm, on the fifth day after labor, *at once and permanently* dissipated all of the unpleasant symptoms. The puerperal fevers are handled very properly.

Our author treats the subject of obstetrical anæsthesia, upon the whole, very judiciously. In no part of his work do we find him lay aside his Scotch prejudices more fully than here. "In eclampsia, in some cases of mania, and in all cases of operative midwifery, it is, without exaggeration, invaluable. In ordinary cases it is always to be used with caution, but if employed in small quantities on a handkerchief on the approach of each pain, towards the termination of the second stage, it can never do harm. It thus allays pain and assuages nervous irritability; and, in the hands of the skilful practitioner, it is a power for good and never for evil."

Heartily do we recommend Leishman's Midwifery to all practicing physicians, but we are sorry to say that we do not find it the text-book for American students which we now feel so much in want of. A new edition of Meigs', from the hands of an able editor, would, we think, be a boon to American students at this time.

